# Authentication of *Cordyceps sinensis* by DNA Analyses: Comparison of ITS Sequence Analysis and RAPD-Derived Molecular Markers

**DOI:** 10.3390/molecules201219861

**Published:** 2015-12-15

**Authors:** Kelly Y. C. Lam, Gallant K. L. Chan, Gui-Zhong Xin, Hong Xu, Chuen-Fai Ku, Jian-Ping Chen, Ping Yao, Huang-Quan Lin, Tina T. X. Dong, Karl W. K. Tsim

**Affiliations:** 1 Division of Life Science, and Center for Chinese Medicine, The Hong Kong University of Science and Technology, Clear Water Bay Road, Hong Kong, China; kellylam@ust.hk (K.Y.C.L.); gallant@ust.hk (G.K.L.C); xingz@cpu.edu.cn (G.-Z.X.); xuhongtcm@yahoo.com.cn (H.X.); yaopingkib@ust.hk (P.Y.); linhuangquan@ust.hk (H.-Q.L.); botina@ust.hk (T.T.X.D.); 2 School of Chinese Medicine, Hong Kong Baptist University, Hong Kong, China; faiku2010@gmail.com; 3 Pharmaceutical Department, Shenzhen Traditional Chinese Medicine Hospital, Guangzhou University of Chinese Medicine, Shenzhen 518033, China; lycjp@126.com

**Keywords:** Cordyceps, *Cordyceps sinensis*, Cordycipitaceae, ITS, RAPD-SCAR, herbal authentication, molecular markers

## Abstract

*Cordyceps sinensis* is an endoparasitic fungus widely used as a tonic and medicinal food in the practice of traditional Chinese medicine (TCM). In historical usage, Cordyceps specifically is referring to the species of *C. sinensis*. However, a number of closely related species are named themselves as Cordyceps, and they are sold commonly as *C. sinensis*. The substitutes and adulterants of *C. sinensis* are often introduced either intentionally or accidentally in the herbal market, which seriously affects the therapeutic effects or even leads to life-threatening poisoning. Here, we aim to identify Cordyceps by DNA sequencing technology. Two different DNA-based approaches were compared. The internal transcribed spacer (ITS) sequences and the random amplified polymorphic DNA (RAPD)-sequence characterized amplified region (SCAR) were developed here to authenticate different species of Cordyceps. Both approaches generally enabled discrimination of *C. sinensis* from others. The application of the two methods, supporting each other, increases the security of identification. For better reproducibility and faster analysis, the SCAR markers derived from the RAPD results provide a new method for quick authentication of Cordyceps.

## 1. Introduction

*Cordyceps sinensis* (Berk.) Sacc. (Fam. Cordycipitaceae), also known as “winter grass and summer worm” (Dong Chong Xia Cao), is a combination of the stroma parasitizing the larva of some species of insects, such as *Hepialus armoricanus* Oberthur of the Hepialidae family. Cordyceps has been used as medicine and a food supplement with an overwhelming list of pharmacological properties for hundreds of years in China. There are about 400 species in the *Cordyceps* genus in the world, mainly distributed in Eurasia, including Java, Sri Lanka, Tasmania, Japanese islands, China and Australia [1,2]. About 60 species are produced in China; more than 30 species are formally reported. According to historical usage, the term Cordyceps normally refers to the species of *C. sinensis*. According to the Chinese Pharmacopoeia 2015, *C. sinensis* is the only source of Cordyceps [3].

Cordyceps is collected in early summer, while the stromata have come up out of the ground, but the spores have not been ejected. Cordyceps is rare and expensive, because it is wild and distributed at an elevation of 3000–5000 m, mainly in the provinces of Yunnan, Sichuan, Qinghai, Xizang and Gansu of China. Due to very limited resources and huge market demand, Cordyceps is the most expensive herbal medicine in China, whose price is comparable to gold [4]. In view of economic value, many producers sold other species of the *Cordyceps* genus as Cordyceps in the market. The substitutes and adulterants of *C. sinensis* are seriously affecting clinical application of this herb. To ensure a continuous efficacy and safety of the herb, permanent quality controls are essential; an important aspect of such control is the verification of species being used as the source material. Therefore, the development of quick and easy methods for the authentication of Cordyceps should have great value.

The known methods in authenticating herbal materials by morphological, microscopic and phytochemical methods are not sufficient in the case of Cordyceps. Independent from environmental influences or origin of raw material, the usage of DNA sequencing or molecular genetic methods can complement the control of authentication [5]. In an attempt to ensure a highly reliable result, two different methods have been developed in authenticating Cordyceps: internal transcribed spacer (ITS) sequences and the random amplified polymorphic DNA (RAPD)-sequence characterized amplified region (SCAR). Both methods are particularly appropriate in dealing with a high percentage of inter-specific sequence divergence, and both approaches have already been successfully used for the identification of medicinal plants in previous studies [6,7,8]. By comparing the outcomes of two methods, the RAPD-SCAR method provided a quicker and user-friendly tool for the authentication of Cordyceps.

## 2. Results

### 2.1. ITS Sequence of Cordyceps

**Figure 1:**
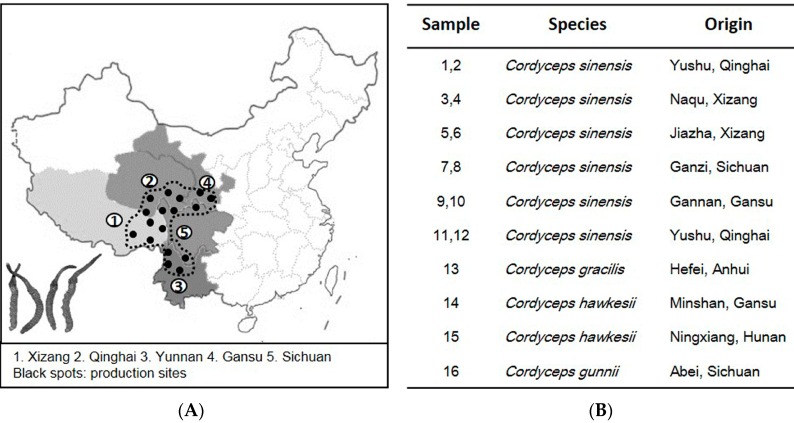
Cordyceps production in China and its collection. (**A**) The geographical locations of major Cordyceps production sites; (**B**) twelve commercial samples of *C. sinensis* (S1–S12) and its adulterants (S13–S16) collected in Hong Kong herbal markets were used in this experiment. Each sample had four batches, *n* = 4.

Figure 1A shows the major production sites of Cordyceps in China. Xizang and Qinghai are considered to produce the best quality herb. In view of these production sites, 12 *C. sinensis* samples were collected, *i.e.*, coded from 1–12 (Figure 1B). The common adulterants, including *C. gracilis*, *C. hawkesii* and *C. gunnii*, were collected, as reference controls. Each species had four different batches (*n* = 4). Different Cordyceps species showed similar morphological appearances, and the authentication was done in-house by using microscope methods according to Chinese Pharmacopoeia 2015.

**Figure 2:**
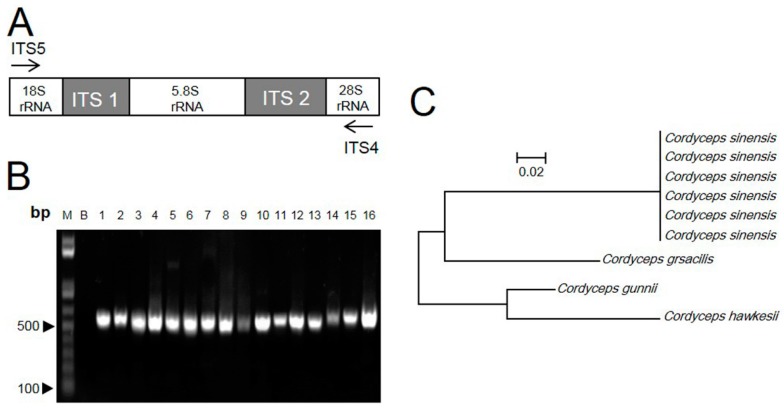
ITS sequence of Cordyceps. (**A**) ITS (internal transcribed spacer) regions and the primers, ITS4 and ITS5, for PCR are shown; (**B**) amplicons of Cordyceps and its adulterants with ITS primers. All samples produced a band at ~550 bp. *n* = 4; (**C**) The ITS sequences were identified and matched fully from sequences of GenBank, *i.e.*, *C. sinensis* (FJ481125), *C. gracilis* (AJ786565), *C. hawkesii* (GU453920) and *C. gunnii* (KJ021181). DNA sequences of ITS fragment from 16 samples were submitted to phylogenetic study by the maximum parsimony method.

The genomic DNA was isolated from dry Cordyceps samples. The procedure yielded about 50–300 ng of DNA from 50 mg of Cordyceps. An absorbance (A260/A280) ratio of 1.8 indicated insignificant levels of contaminating proteins and polysaccharides. For the identification of the ITS sequence, two flanking primers, ITS4 and ITS5, were used for PCR (Figure 2A). About 580 bp were amplified and sequenced from all samples (Figure 2B). Based on the nucleotide sequence analysis, including the complete ITS1, ITS2 and 5.8S rRNA region, the ITS sequences of *C. sinensis* were highly homologous, regardless of geographical origin. In contrast, the sequences from *C. gracilis, C. hawkesii* and *C. gunnii* were highly divergent from *C. sinensis*, and the distance values ranged from 0.25–0.33 (Figure 2C). Thus, the rRNA ITS region should be able to distinguish Cordyceps at the DNA level.

### 2.2. RAPD-SCAR Application in Cordyceps

**Figure 3:**
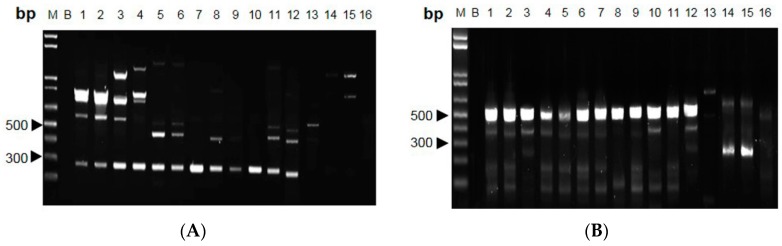
Profiles of Cordyceps species amplified with RAPD primers. (**A**) AP-G 05 and (**B**) AP-I 07 were used for PCR, and the products were subjected to 1.2% agarose gel. Lane M: 1-kb DNA ladder; Lane B: blank (nuclease-free distilled water); Lanes 1–12: *C. sinensis*; Lane 13: *C. gracilis*; Lanes 14–15: *C. hawkesii*; Lane 16: *C. gunnii*. Independent experiments were repeated three times, *n* = 3.

The polymorphic DNA patterns of *C. sinensis* were screened from 68 arbitrary decamer primers [9]. Five RAPD primers, for which variability and applicability were tested in preliminary experiments, were chosen for Cordyceps DNA analysis. With these primers, all specimens showed highly repeatable patterns with the finally chosen protocol and reaction conditions (data not shown). The primer AP-G 05 and AP-I 07 consistently amplified intense bands of ~290 and ~540 bp, respectively, for *C.* *sinensis* (Figure 3A,B). This *C. sinensis*-specific amplified band was absent in other *Cordyceps* species.

**Figure 4:**
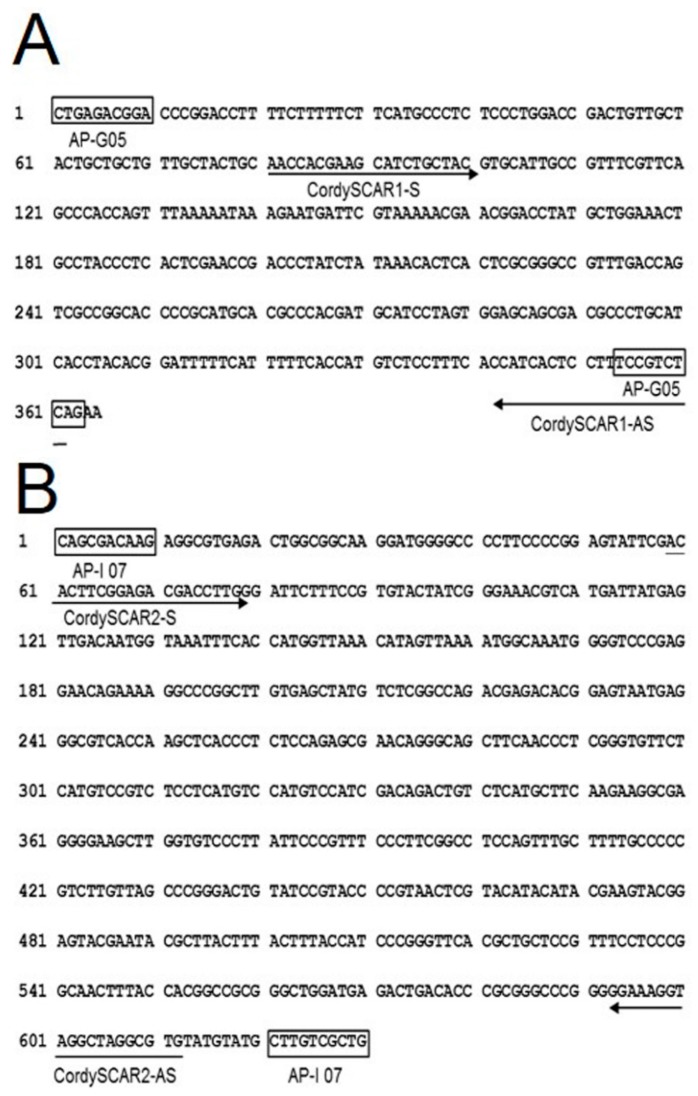
Nucleotide sequence of the RAPD amplicon. (**A**) DNA sequence of the PCR product generated from AP-G 05. RAPD primers (a pair of AP-G 05) and SCAR primers (CordySCAR1-S and CordySCAR1-AS) were indicated; (**B**) DNA sequence of the PCR product generated from AP-1 07. RAPD primers (a pair of AP-I 07) and SCAR primers (CordySCAR2-S and CordySCAR2-AS) were indicated.

Both AP-G 05 and AP-I 07 amplified bands were sub-cloned and sequenced (Figure 4A,B). The first ten nucleotides obtained matched completely with the corresponding RAPD primers used. This result clearly showed that the cloned fragment was derived from the amplified RAPD product. The length of the AP-G 05 marker obtained was 365 bp with 52% G + C content (A: 81; C: 123; G: 67; T: 94), and the length of AP-I 07 marker obtained was 630 bp with 54.8% G + C content (A: 135; C: 174; G: 171; T: 150).

**Figure 5:**
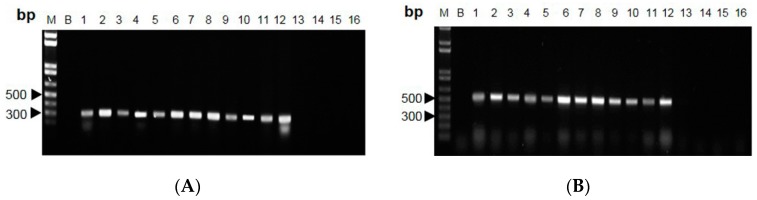
PCR amplification of *Cordyceps* species using SCAR primers. (**A**) CordySCAR1 and (**B**) CordySCAR2 were used for PCR, and the products were subjected to 1.2% agarose gel. Lane M: 1-kb DNA ladder; Lane B: blank (nuclease-free distilled water); Lanes 1–12: *C. sinensis*; Lane 13: *C. gracilis*; Lanes 14–15: *C. hawkesii*; Lane 16: *C. gunnii.* Independent experiments were repeated three times, *n* = 3.

The designed SCAR primer pair was used to amplify genomic DNA from the twelve *Cordyceps* species (Figure 4). Distinct bands of 291 bp and 554 bp were obtained in DNA isolated from all *C*. *sinensis* species (*i.e*., S1–S12), and no non-specific amplification was observed in other *Cordyceps* species (*i.e.*, S13–S16) (Figure 5A,B). In addition, the designed primers were employed to authenticate the Cordyceps herbs collected in the Hong Kong market, and out of 100 samples that we tested, none of them failed (data not shown). Thus, the RAPD-SCAR method by using the designed primers could be able to distinguish *Cordyceps* species.

## 3. Discussion

Traditional Chinese medicine (TCM) has made contributions to the health of the Chinese population for several thousand years, as well as the development of herbal medicine worldwide, leaving memorable glimpses in the history of world medicine. However, many erroneous substitutes and adulterants of TCM are traded in the global market due to their lower costs or misidentification of species with very similar morphological features. Substitutes and adulterants of TCM materials may have no therapeutic effects and/or, at the worst, lead to poisoning. Identification of TCM by its morphology is time consuming and labor intensive. Identification of TCM by DNA analysis is one of the most reliable methods, which is not affected by age, physiological conditions, environmental factors, as well as the methods of harvest, storage and processing [10].

Two different DNA-based approaches, ITS sequencing and RAPD-SCAR, for the authentication of Cordyceps were employed here. The ITS regions are the routine markers in evolutionary studies at different taxonomic levels due to different rates of evolution [11,12]: this is widely used in the identification of medicinal plants, e.g., *Astragalus membranaceus* [13], *Adenophora stricta* [14], *Angelica anomala* [15], *Boerhavia diffusa* [16] and *Lonicera japonica* [17]. In contrast, RAPD analysis can reveal a high degree of polymorphism, which does not require prior DNA sequence information, and more importantly, this is easy to perform. Therefore, many researchers have explored its application for the authentication of medicinal plants, e.g., *Crocus sativus* [18], *Panax notoginseng* [19], *Panax* *ginseng* [20] and *Fritillariae cirrhosae* [21]. In our study, both ITS and RAPD-SCAR DNA markers could distinguish Cordyceps and its adulterants *C. gracilis*, *C. hawkesii* and *C. gunnii*. The RAPD-SCAR method meets the urgent needs of the present herbal market, which may be used to screen a large number of Cordyceps batches before their grading, as to exclude those adulterants in the market. The availability of genetic sequences could allow the development of technological devices, such as gene chips or specific kits, to be enforced in routine control.

**Figure 6:**
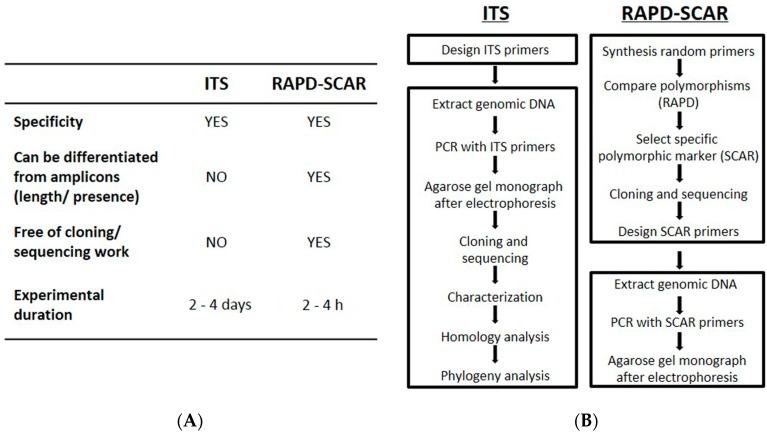
Comparison of ITS sequences and the RAPD-SCAR method. (**A**) The characteristics of the ITS and RAPD-SCAR methods; (**B**) workflow of the ITS and RAPD-SCAR methods.

Both ITS sequences and the RAPD-SCAR marker enabled discrimination of *C. sinensis* from its adulterants. The corroborative results of these two methods were totally coincidental. The basic features of each method are compared (Figure 6A). The RAPD-SCAR is a simple, accurate and reliable method in identifying *C. sinensis* at the DNA level, which includes: (i) eliminating the problem in DNA sub-cloning and sequencing; (ii) shortening the experimental time; (iii) minimizing sample size; and (iv) providing a yes or no conclusion. In addition, the possible errors that could happen in ITS sequencing are markedly reduced: because the individual just has to run one PCR from the newly-developed RAPD-SCAR method (Figure 6B). The SCAR markers have advantages for largescale analyses because of the low cost, high reproducibility and just a simple PCR reaction.

## 4. Materials and Methods

Different species of Cordyceps were collected in Hong Kong and Mainland China, and the specimens were identified by the authors; the voucher specimens were deposited in the Center for Chinese Medicine of Hong Kong University of Science and Technology. Twelve samples of *C. sinensis* (S1–S12) and its adulterants (S13–S16) were collected. Four batches (each at ~20 g) were collected from each sample.

### 4.1. DNA Extraction

For DNA extraction, Cordyceps was ground into a fine powder with a grinder. The extraction was then performed with a commercial kit (DNeasy^®^Plant Mini Kit, Qiagen Hilden, Germany) according to the manufacturer’s protocol. The DNA quantification was done with a NanoDrop 2000 Spectrophotometer (Thermo Fisher Scientific Inc., Waltham, MA, USA).

### 4.2. ITS Analysis

For a 50-μL PCR reaction, 5 μL of genomic DNA (~50 ng) were added to a master mix containing 10× PCR buffer (with Mg^2+^ at 1× concentration of 1.5 mM and containing loading dye), 0.5 m M dNTP, 400 nM forward and reverse primers (ITS5: AGG AGA AGT CGT AAC AAG and ITS4: GTT TCT TTT CCT CCG CT) [2] and 1.5 U of Taq Polymerase (KAPA Taq DNA Polymerase with dye, KAPA Biosystems, Woburn, MA, USA). A GeneAmp 9700 thermal cycler (Applied Biosystems, Foster City, CA, USA) was programmed with 3 min at 95 °C, 30 s at 55 °C and 45 s at 72 °C, followed by 40 cycles of 30 s at 95 °C, 30 s at 55 °C and 30 s at 72 °C; the final extension was 10 min at 72 °C. An aliquot (5 μL) of the amplification product was separated on a 1.2% agarose gel and detected under UV-light after staining in SYBR^®^ Safe DNA gel stain (Thermo Fisher Scientific Inc.). The PCR product was purified with a ready-to-use PCR purification kit (Qiagen) before sequencing. The cycle sequencing, as well as the sequence reaction itself were performed by an external company (Dragon Technology Limited, Hong Kong, China). All sequences were edited with Bioedit and aligned with the ClustalW algorithm of the Molecular Evolutionary Genetics Analysis version 6.0 (MEGA 6) software (Biodesign Institute, Arizona, MA, USA). All sequence distances were calculated with MEGA 6.

### 4.3. RAPD Analysis

PCR for amplification conditions was optimized to increase the reproducibility of the banding pattern. The RAPD was carried out with 5 μL of genomic DNA (~50 ng) in a 50-μL reaction containing 10× PCR buffer (with Mg^2+^ at a 1× concentration of 1.5 mM and containing loading dye), 0.5 mM dNTP, 400 nM random primers and 1.5 U of Taq Polymerase (KAPA Biosystems). Five random primers, AP-A 20 (5’-GTT GCG ATC C-3’), AP-D 18 (5’-GAG AGC CAA C-3’), AP-G 05 (5’-CTG AGA CGG A-3’), AP-H 18 (5’-GAA TCG GCC A-3’) and AP-I 07 (5’-CAG CGA CAA G-3’), were finally employed: because they produced bands for all samples. The PCR was carried out with a GeneAmp 9700 thermal cycler. The cycling conditions consisted of an initial 5 min at 95 °C followed by 1 min denaturing at 94 °C, 1 min annealing at 36 °C and 2 min elongation at 72 °C, repeated 40 cycles, and with 5 min of final extension at 72 °C. An aliquot (5 μL) of the amplification product was separated on a 1.2% agarose gel. The PCR products were sub-cloned and sequenced. In the amplification of genomic DNA by SCAR primers, the designed primers were used for *Cordyceps* species. Thermal cycling conditions for amplification using SCAR primers were optimized as: 95 °C for 5 min; 28 cycles at 95 °C for 1 min, 65 °C for 1 min and 72 °C for 1 min; and a final extension at 72 °C for 10 min. Identification of PCR was done by a gel electrophoresis.

### 4.4. RAPD Amplification and SCAR Design

The PCR bands amplified by the random primer (AP-G 05) and (AP-I 07) were excised from 1.2% agarose gel with a sterile gel slicer and purified using the QIAquick Genei Gel Extraction kit (Qiagen). TA cloning strategy was employed using the Invitrogen TA Cloning system (Invitrogen, Carlsbad, CA, USA). Transformation was carried out using high efficiency competent cells (DH-5α strain of *Escherichia coli*) following the protocol for transformation by calcium chloride, as described by Sambrook *et al.* [22]. Ten distinct white colonies were picked up from the LB-ampicillin plate. Then, the recombinant plasmid DNA from *E. coli* was isolated using Qiagen’s QIAprep^®^ Mini prep kit following the manufacturer’s directions. Confirmation of the clones was done by sequencing the DNA using M13 and T7 universal primers. After the purified RAPD amplicon was cloned, both ends of the recombinant plasmid were sequenced on an ABI 3700 automated sequencer (Applied Biosystems). Homology searches were performed within GenBank’s non-redundant database using the BLAST algorithm available at http://www.ncbi.nlm.nih.gov/BLAST/ [23]. Based on the sequenced RAPD amplicon, two pairs of SCAR primers (CordySCAR1 and CordySCAR2) amplified 291 bp and 554 bp from Cordyceps DNA.

## 5. Conclusions

Our study suggests that the usage of RAPD-derived DNA markers in authenticating *C. sinensis* could be helpful in ensuring the quality of Cordyceps. Additionally, this method could facilitate the testing and certification of other herbal materials in the pharmaceutical industry.
